# First Molecular Characterization of Small Ruminant Lentiviruses Detected in Romania

**DOI:** 10.3390/ani13233718

**Published:** 2023-11-30

**Authors:** Monika Olech, Dragoş Hodor, Corina Toma, Andrada Negoescu, Marian Taulescu

**Affiliations:** 1 Department of Pathology, National Veterinary Research Institute, 24-100 Puławy, Poland; 2 Department of Veterinary Pathology, University of Agricultural Sciences and Veterinary Medicine, 3-5 Calea Manastur, 400372 Cluj-Napoca, Romania; dragos.hodor@usamvcluj.ro (D.H.); corina.toma@usamvcluj.ro (C.T.); andrada.negoescu@usamvcluj.ro (A.N.); marian.taulescu@usamvcluj.ro (M.T.)

**Keywords:** SRLV, Maedi-visna virus, MVV, phylogenetic analysis, nested real-time PCR

## Abstract

**Simple Summary:**

Small ruminant lentiviruses (SRLVs) are a group of highly diverse viruses responsible for global infections in goats and sheep. The purpose of this study was to genetically characterize SRLVs circulating in Romania. A total of 122 samples from pulmonary and lung lymph nodes of slaughtered sheep were examined. The obtained *gag-pol* and *gag* sequences from Romanian SRLV strains were compared with available GenBank strains, and the results showed that the Romanian sequences were associated with strains A2 and A3 based on *gag-pol* sequences and with subtypes A3 and A17 based on *gag* sequences. In addition, the Romanian sequences showed some specific mutations in epitope 3, which may reflect their evolution. This study describes for the first time SRLV sequences detected in Romanian sheep, providing basic information on the subtypes circulating in Romania.

**Abstract:**

Small ruminant lentiviruses (SRLVs) are a group of retroviruses that cause multisystem chronic diseases in goats and sheep and lead to production losses in these animals, negatively affecting animal health and welfare. Although molecular characterization of SRLV field isolates has been performed in many countries, there is currently no information on SRLV genotypes circulating in sheep and goats in Romania. Therefore, the main objective of this study was to conduct a molecular and phylogenetic analysis of SRLVs from Romania and determine the degree of genetic relatedness of the obtained sequences to other known SRLV reference strains. A total of 81 sheep lung tissue samples and 41 sheep lung lymph node samples were tested using nested real-time PCR, and samples positive for real-time PCR were used to amplify an 800 bp *gag-pol* fragment and an overlapping 625 bp fragment of the *gag* gene. Pairwise DNA distance and phylogenetic analysis showed that the Romanian SRLV strains were closely related to the A2 and A3 strains based on *gag-pol* sequences and to the A3 and A17 subtypes based on *gag* sequences. No recombination events were found. Our results revealed that the Romanian sequences have similar epitope patterns to other existing subtypes, although E/K and R/K mutations in epitope 3 were found only in the Romanian sequences, which may have potential value in serological diagnosis. This study is the first report on the genetic characterization of SRLV strains circulating in Romania and provides new information on SRLV heterogeneity. Further detailed studies should be conducted to better understand the divergence of SRLV Romanian strains.

## 1. Introduction

Small ruminant lentiviruses (SRLVs) are highly heterogeneous retroviruses belonging to the genus *Lentivirus* in the family *Retroviridae* [1]. SRLVs include two related viruses, Maedi-visna virus (MVV) and Caprine arthritis encephalitis virus (CAEV), which can infect both sheep and goats, as these viruses can cross the species barrier. SRLVs cause a multisystem disease with progressive and persistent inflammatory changes in the mammary gland, lungs, joints and brain. SRLVs cause latent infections, and most infected animals are clinically healthy. After a latent period, which can last several years, about one-third of infected animals develop signs such as pneumonia, arthritis, mastitis, encephalitis and weakness. There are no effective drugs or vaccines for these viruses, and infection is usually controlled through serological testing and elimination of infected animals. Due to the significant economic impact of SRLVs, MV (Maedi-visna) and CAE (Caprine arthritis encephalitis) have been included on the OIE list, and many countries have eradication and control programs [2].

The SRLV genome consists of two linear molecules of single-stranded RNA that are converted to double-stranded (ds) DNA via the viral enzyme reverse transcriptase (RT), and then the viral genome is integrated into the host genome as a provirus. SRLVs are classified as so-called complex retroviruses because their genome contains genes that encode structural proteins and enzymes (*gag*, *pol* and *env*) and auxiliary genes that contain information on the synthesis of proteins that regulate viral replication (*vpr-like*, *rev* and *vif*). The integrated proviral DNA is flanked by non-coding sequences called long terminal repeats (LTRs), which contain regulatory elements necessary for proviral integration, transcription and polyadenylation of viral RNA [3].

Genetic variability is the main feature of SRLVs. SRLVs occur in individual animals as a population of genetic variants, quasi-species that are continuously generated by mutations, mainly due to the low fidelity of reverse transcriptase, recombination and selection pressure by the host immune system [3]. A number of studies have been conducted to investigate the phylogeny and genetic variability of partial or complete sequences of SRLV field isolates from different geographic regions. Phylogenetic analyses have been conducted based on *gag*, *pol*, *env* and LTR sequences [4,5,6,7,8,9]. The *gag* and *pol* genes are relatively well conserved among SRLVs, making them ideal targets for designing PCR primers. Currently, the classification of SRLVs is carried out mainly on the basis of the conserved *gag* fragment encoding the capsid protein, for which sequences representing almost all subtypes are available. To date, SRLVs have been divided into five groups (A–E), which vary 25–37% in nucleotide sequences. Groups A, B and E are further subdivided into different subtypes (A1–A27, B1–B5 and E1–E2) [10,11]. However, as more and more local strains are analyzed, new subtypes are constantly emerging, indicating the continued need for surveillance of diagnostic strategies.

MVV and CAEV are prototypes of groups A and B, respectively, and are widely distributed in sheep and goat populations worldwide. The other three groups are less common and are restricted to specific geographic areas. Groups C and E have been isolated in Norway and Italy, respectively, while genotype D was restricted to Switzerland and Spain [11]. Information on the SRLV subtypes circulating in each country is important for monitoring antigenic variation, since antigenic variation can be responsible for misdiagnosis of highly divergent strains [12].

Although molecular characterization of SRLV field isolates has been conducted in many countries, there is currently no information on SRLV genotypes circulating in sheep and goats in Romania. Therefore, the main objective of this study was to conduct a molecular and phylogenetic analysis of SRLVs from Romania and to determine the degree of genetic relatedness of the obtained sequences to other known SRLV reference strains. This is the first report describing the genetic characteristics of SRLVs identified in Romania.

## 2. Materials and Methods

### 2.1. Samples

**Figure 1:**
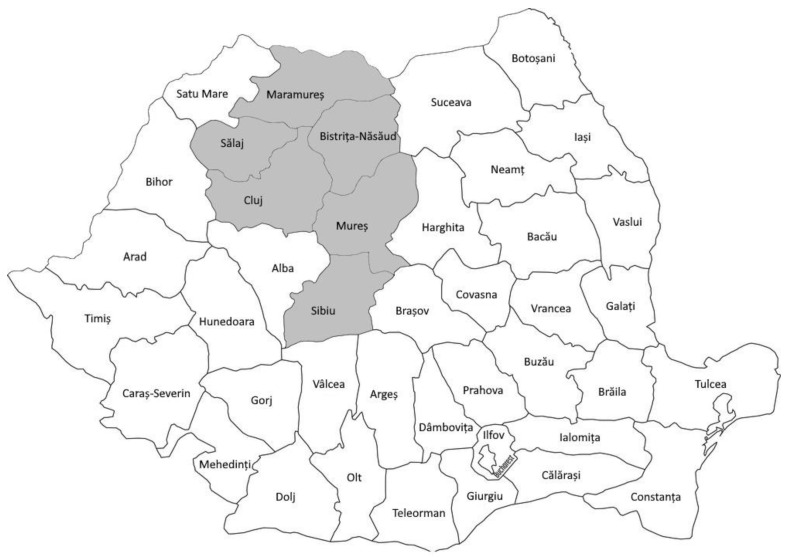
Map of Romania. The geographical distribution of SRLV samples analyzed in this study are marked in gray.

A total of 81 lung tissue samples and 41 pulmonary lymph nodes samples were collected at the slaughterhouse from Turcana sheep showing chronic pulmonary inflammatory lesions on macroscopical examination. Both lung tissue and lung lymph nodes were collected from 41 animals, while only lung tissue samples were collected from 40 animals. The samples came from 6 counties (Bistrita-Nasaud, Sibiu, Maramures, Mures, Salaj and Cluj) located in northwestern and central Romania (Figure 1). Since the samples were taken from dead animals, ethical approval was not required. The samples were collected between 2017 and 2022. Ethical review and approval was not required for the study because no experimental procedures were performed on animals.

### 2.2. DNA Extraction

DNA was obtained from 25 mg of each sample using the Nucleospin Tissue kit (Macherey Nagel GmbH & Co. KG, Duren, Germany). Finally, DNA was eluted in 100 µL of elution buffer following the manufacturer’s instructions. The quality and quantity of DNA were assessed in a nanophotometer (Implen, Munich, Germany).

### 2.3. Nested Real-Time PCR for the Proviral Detection of SRLVs

The nested real-time PCR was performed as previously described by Schaer et al. [13] with slight modifications. The first step consisting of a conventional PCR was performed using Thermal Cycler (Biometra, Göttingen, Germany). Reaction included 2U of OptiTaq DNA Polymerase (EURx, Gdańska, Poland), 1× PCR buffer with 1.5 mM MgCl_2_, 300 nM of each primer, 0.2 mM of dNTP-mix and 1 µg of extracted DNA. Amplification was performed in a total volume of 25 μL according to the following cycling conditions: initial denaturation at 95 °C for 5 min, followed by 40 cycles of denaturation at 94 °C for 30 s, annealing at 60 °C for 30 s and elongation at 72 °C for 1 min and final elongation at 72 °C for 10 min. All products of the first PCR were then tested in second step with genotype-specific real-time PCRs using primers and probes specific for detection and discrimination of genotypes A and B of the SRLV. The qPCR was performed on 7500 Fast Real-time PCR system machine (Applied Biosysteme, Foster City, CA, USA). The reaction mixture for each PCR test contained 10 μL 2× QuantiTect Probe PCR Master Mix (Qiagen, Venlo, The Netherlands), 900 nM of each primer, 200 nM of the specific probe and 5 μL of PCR product of the first step. Amplification profiles consisted of a hold stage of 15 min at 95 °C and PCR stage of 40 cycles at 94 °C for 15 s and 60 °C for 60 s. A no-template control (NTC) consisting of deionized H_2_O was prepared as a negative control and included in each run. All tested samples were tested with primers and probes designed for detection of MV- and CAE-like viruses.

### 2.4. PCR Amplification, Sequencing and Sequence Analysis

Samples, which were nested real-time PCR positive, were selected for amplification of the 800 bp *gag-pol* fragment and overlapping 625 bp fragment of the *gag* gene. Nested PCR protocols were used for amplification of these two genomic fragments, as previously described [14,15]. A water template negative control was run parallel with each PCR reaction set. PCR products of the second PCR were purified and directly sequenced in both directions by the Genomed SA Company (Warsaw, Poland) using 3730 xl DNA Analyzer (Applied Biosystems, Foster City, CA, USA) and a BigDye Terminator v3.1 Cycle Sequencing kit. The obtained SRLV sequences were manually checked and edited using Geneious Pro 5.3 software (Biomatters Ltd., Auckland, New Zealand). Nucleotide sequences were aligned using the Clustal W algorithm with SRLV reference strains retrieved from GenBank. The GTR statistical model with gamma distribution (G) and invariant sites (I) was used as the best-fitting model to create a phylogenetic tree using the maximum likelihood (ML) method. Neighbor-joining tree was constructed using Tamura–Nei model. The robustness of the clusters was assessed by performing 1000 bootstrap repetitions. Alignment, model testing and tree building were performed using MEGA 6 application [16]. The pairwise genetic distances between samples and reference strain sequences were estimated with the p-distance model applying the gamma distribution parameter using MEGA 6 software.

All novel sequences obtained in this study were submitted to the GenBank database under accession numbers: OR666866-OR666886 for the *gag* sequences and OR671958–OR671978 for the *gag-pol*.

### 2.5. Analysis of Recombination

Recombination Detection Program version 4 (RDP4) was used to identify potential recombination events and recombination breakpoints using seven methods (RDP, GENECONV, BootScan, MaxChi, Chimaera, SiScan and 3Seq) implemented in RDP4 package software [17]. Putative recombinant events were considered significant when *p* ≤ 0.01 was observed for the same event using four or more methods.

## 3. Results

### 3.1. Amplification and SRLV Sequences

Out of 122 samples tested, 63 were positive via nested real-time PCR. A total of 40 of these samples derived from the lungs, while 23 samples derived from pulmonary lymph nodes. All 63 samples were positive only with primers and a probe specific for the MVV. The CAEV was not detected. All positive samples were then used to amplify the *gag-pol* fragment (800 bp) and the overlapping *gag* fragment (625 bp) encoding the capsid protein. A total of 31 samples (18 from lung and 13 from lung lymph nodes) were successfully amplified using *gag-pol* primers, while 24 samples (12 from lung and 12 from lung lymph nodes) were successfully amplified using *gag* primers. The 28 samples that yielded a strong *gag-pol* PCR product were sequenced, and 21 good-quality sequences were obtained. For five samples, reliable sequences were not obtained due to high background. For the *gag* fragment, 21 of 24 sequences were obtained. For four samples, only *gag* sequences were obtained, and for four samples, only *gag-pol* sequences were obtained. For 17 samples, both *gag-pol* and *gag* sequences were obtained (Table 1). For the latter samples, *gag* and *gag-pol* gene sequences were compared and showed 100% identity in the overlapping *gag* region.

### 3.2. Phylogenetic Analysis of SRLV Strains Based on Gag-Pol Fragment

A total of 21 partial *gag-pol* SRLV sequences were aligned to each other and to reference strains representing group A (subtypes A1, A2, A3, A2/A3, A4, A5, A7, A8, A9, A11, A19, A20, A21, A22, A23 and A24), B (subtypes B1, B2 and B3), C and E (subtypes E1 and E2). Phylogenetic analysis was performed using NJ and ML and led to the same classification of strains. Results of this study revealed that all SRLV sequences isolated from sheep from Romania belonged to genotype A. All sequences were fairly homogeneous. The mean degree of genetic variation in the Romanian sequences was 8.9% with a range of variation from 0% to 13.2%. The sequences #RO43_Lymph node, #RO27_Lung and #RO35_Lymph node showed 100% homology. In addition, sequences #RO46_Lymph node, #RO36_Lung and #RO26_Lung and sequences #RO42_Lymph node and #RO20_Lung showed 100% sequence identity.

**Figure 2:**
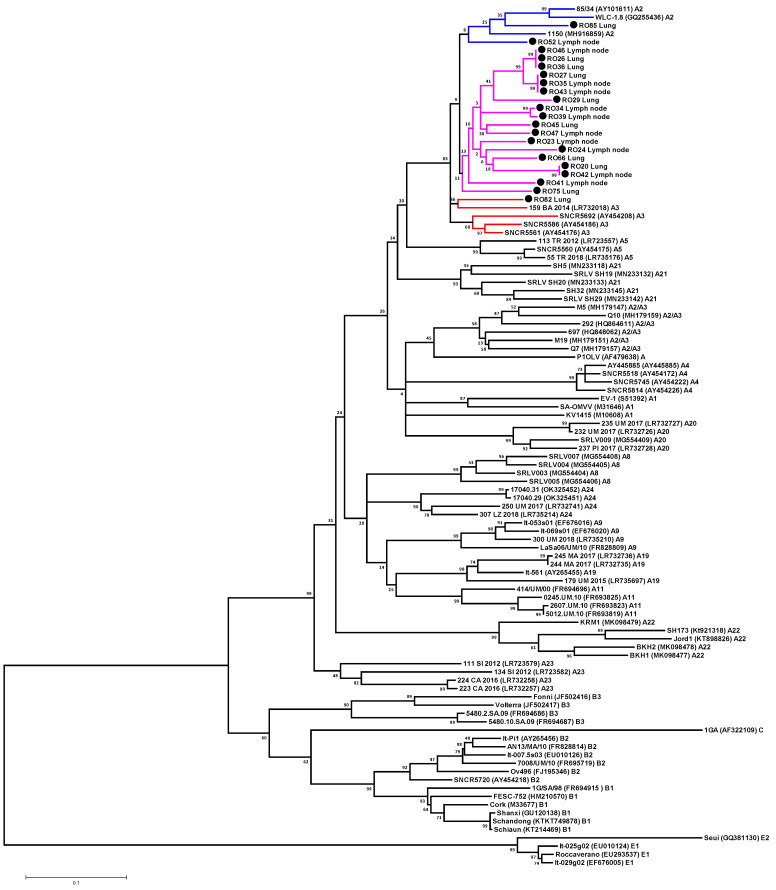
Unrooted maximum likelihood phylogenetic tree based on the alignment of 647 nt from *gag-pol* region of 98 sequences: 21 analyzed in this study (labeled by a black circle) and 83 reference strains available in GenBank. Scale bar: number of substitutions per site. The numbers on the nodes indicate the percentage of bootstrap values obtained from 1000 replicates.

The assignment of Romanian sequences to specific subtypes was unclear. Sample #RO82 isolated from the lung clustered together with reference strains belonging to subtype A3, but the genetic distance between this sample and sequences belonging to subtypes A3 and A2 was similar, ranging from 9.5% to 11.4% and from 11.1% to 13%, respectively (Figure 2, Table S1). Samples #RO52_Lymph node and #RO85_Lung formed a cluster with reference strains belonging to subtype A2 but without significant statistical support. Moreover, the genetic distance between these samples and sequences belonging to subtypes A2 and A3 was also similar and ranged from 10.1% to 11.4% and from 8.2% to 13%, respectively (Figure 2, Table S1). Therefore, a clear genotype determination was impossible. The remaining 19 Romanian sequences formed a single cluster, but without significant statistical support, and the similarity values of these sequences were intermingled between the A2 and A3 subtypes. The mean nucleotide divergence between these 19 Romanian sequences and those representing subtypes A2 and A3 was 12.2% and 11.6%, respectively. Therefore, all Romanian isolates based on *gag-pol* fragment should be classified as A2/A3. Moreover, Romanian sequences did not cluster together with the A2/A3 sequences described so far, indicating that these sequences are different. These results were confirmed using the pairwise distances comparison, as the mean genetic distance of Romanian sequences and known A2/A3 sequences was 15.6% (Figure 2, Table S1). No recombination events were observed for the Romanian *gag-pol* sequences based on RDP analysis. 

### 3.3. Phylogenetic Analysis of SRLV Strains Based on Gag Fragment

To determine relationship between the Romanian sequences and other SRLV sequences, ML and NJ phylogenetic trees based on *gag* sequence alignment were constructed. A total of 25 Romanian partial SRLV *gag* sequences were aligned to each other and to reference strains representing groups A (subtypes A1–A5, A7–A9, A11–A13 and A16–A27), B (subtypes B1, B2 and B3), C and E (subtypes E1 and E2). Sequences representing almost all subtypes detected to date were used for analysis. Only sequences representing subtypes A6, A10, A15 and B5 were not included, as only *pol* fragments of these subtypes were available. In addition, sequences representing subtypes A14 had to be excluded from analyses due to the shortness of the corresponding sequence fragment. Subtype B4 was also excluded because it appeared to be a recombinant strain [18]. Due to the detection of new subtypes at the same time, in the present study, the SRLV subtypes detected by Colitti et al. [19] were renamed from A18 to A19 and from A19 to A20 and the subtypes detected by Olech et al. [5] were renamed from A23 and A24 to A25 and A26, respectively. 

**Figure 3:**
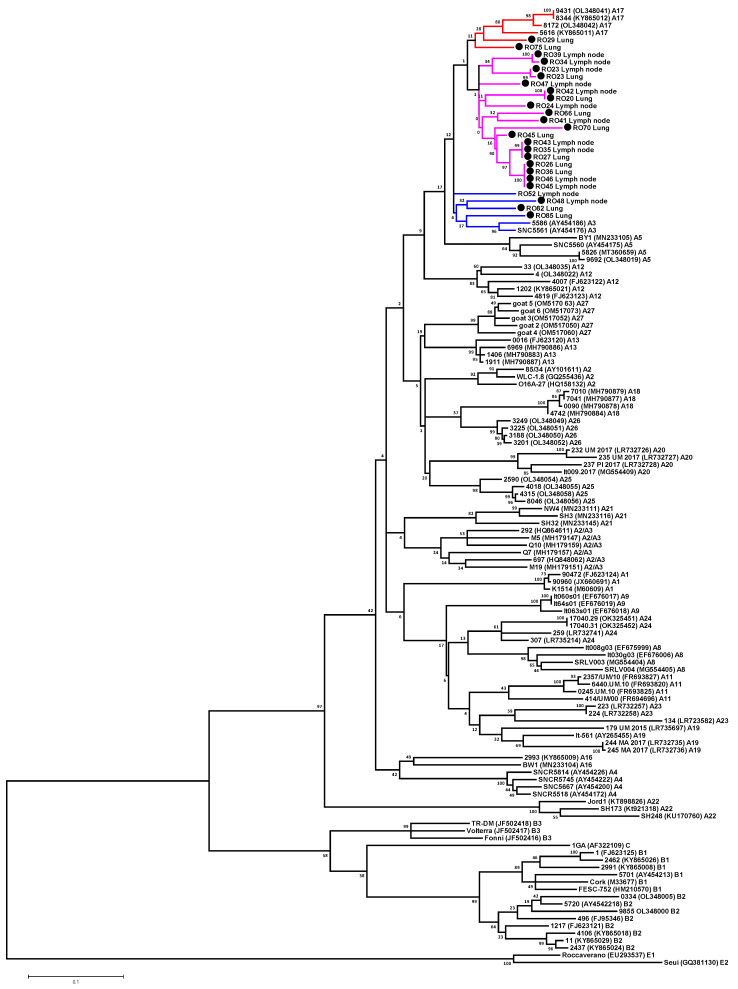
Unrooted maximum likelihood phylogenetic tree based on the alignment of 420 nt from *gag* region of 134 sequences: 25 analyzed in this study (labeled by a black circle) and 109 reference strains available in GenBank. Scale bar: number of substitutions per site. The numbers on the nodes indicate the percentage of bootstrap values obtained from 1000 replicates.

The 25 Romanian *gag* sequences analyzed in this study included 21 sequences obtained by amplification of the *gag* fragment and 4 sequences derived from the *gag-pol* fragment. Phylogenetic analysis was performed using NJ and ML and led to the same classification of strains. Phylogenetic analysis revealed that all sequences from Romanian sheep belonged to genotype A (Figure 3). The mean genetic similarity between Romanian sequences was 8.3% and varied from 0% to 12.8%. Sequences #RO26_Lung, #RO36_Lung, #RO45_Lymph node and #RO46_Lymph node were identical. Furthermore, sequences #RO27_Lung, #RO35_Lymph node, #RO43_Lymph node as well as sequences #RO20_Lung and RO42_Lymph node showed 100% sequence identity. Phylogenetic trees revealed that the sequences #RO52_Lymph node, #RO82_Lung, #RO85_Lung and #RO48_Lymph node were placed close to the subtype A3 sequences, but with no significant statistical support. The mean genetic distance of these sequences and those representing subtype A3 ranged from 9.4% to 9.9%. These sequences were also closely related with A17 strains. The mean genetic variability between these sequences ranged from 9.8% to 13.2%. The #RO29_Lung and #RO75_Lung sequences clustered with sequences representing subtype A17, but this cluster was formed without significant statistical support. Sequence #RO29_Lung was most closely related to the A17 strains, showing a mean genetic distance of 9.2%. Sequence #RO75_Lung showed equal similarity to the A3 and A17 subtypes with mean genetic distances of 9.7% and 9.6%, respectively. The remaining 19 Romanian sequences formed a single cluster that was not supported by the bootstrap value and showed equal similarity to sequences representing subtypes A3 and A17. The mean genetic distances between these 19 Romanian sequences and sequences belonging to subtypes A3 and A17 ranged from 8.3% to 11.4% and from 8.3% to 11.4%, respectively. Therefore, all these Romanian isolates should be classified as A3/A17 based on the *gag* fragment. Results are shown in Table 2 and Figure 3. No sequences clustered with strains belonging to genotypes B, C, D and E and no recombination events were detected. 

### 3.4. Comparative Analysis of Immunodominant Regions

**Figure 4:**
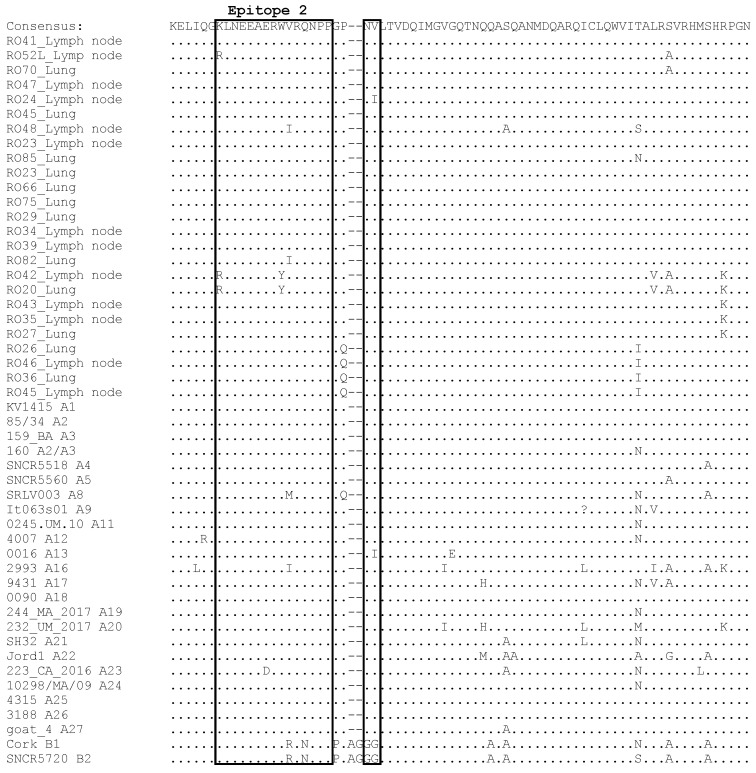

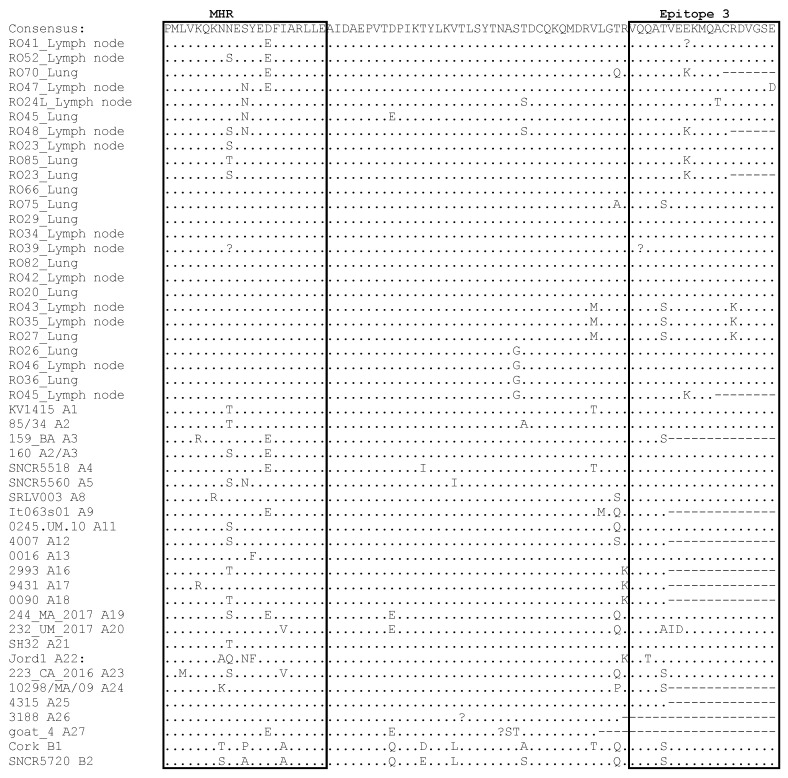
Amino acid sequence multiple alignment of SRLVs deduced from the *gag* fragment. Romanian sequences have been aligned with the reference strains representing known subtypes of genotypes A and B. Immunodominant epitopes 2 and 3, Major Homology Region (MHR) and asparagine-valine (NV) motif are within squares. Dots represent the same amino acid residue.

The nucleotide sequences of *gag* were translated into amino acid sequences, and the results of the alignment and comparison with the most representative sequences representing known subtypes of genotypes A and B are shown in Figure 4. Specifically, immunodominant regions in SRLV sequences contain epitopes 2 and 3, double glycine (GG) motif and Major Homology Region (MHR). Although there was moderate nucleotide heterogeneity, the amino acid sequences of the Romanian strains were conserved because nucleotide mutations were synonymous. All Romanian sequences showed the presence of an asparagine-valine (NV) motif specific for all SRLV’s genotype A. Sequences of epitope 2 showed a high degree of conservation. Only lysine (K) was replaced by arginine (R) in samples #RO52_Lymph node, #RO42_Lymph node and #RO20_Lung, tryptophan (W) was replaced by tyrosine (Y) in samples #RO42_Lymph node and #RO20_Lung and valine (V) was replaced by isoleucine (I) in sample #RO48_Lymph node. More alterations were found in epitope 3, where 11 out 25 Romanian samples showed changes. Specifically, the replacement of glutamic acid (E) with lysine (K) was observed in five samples, the replacement of threonine (T) with serine (S) was observed in four samples, the replacement of arginine (R) with lysine (K) was observed in three samples and the replacement of alanine (A) with threonine (T) and the replacement of glutamic acid (E) with aspartic acid (D) was observed in one sample. The substitutions of glutamic acid (E) by lysine (K) and arginine (R) by lysine (K) were observed only in some Romanian sequences, while the substitution of threonine (T) by serine (S) was also observed in sequences representing subtypes A3, A23, A24, B1 and B2. Some changes were also observed in the Major Homology Region. Four Romanian sequences had glutamic acid (E) instead of aspartic acid (D), four sequences had asparagine (N) instead of serine (S), four sequences had serine (S) instead of asparagine (N) and one sequence had threonine (T) instead of asparagine (N). D/E, S/N and N/S, T mutations were also observed in sequences representing subtypes A3, A2/A3, A4, A9, A19 and A27, subtypes A5, A22 and A1, A2, A2/A3, A5, A11, A12, A16, A18, A19, A21 and A23, respectively.

## 4. Discussion

SRLVs have a significant economic effect on small ruminant livestock production, but their impact on goat and sheep production is underestimated. No treatment or vaccine has been developed against SRLVs. Therefore, control and eradication programs are the only way to avoid the spread of SRLV infection. However, most countries do not pay much attention to SRLV infection control, and eradication programs are implemented in countries where goats and sheep are extensively reared [20,21,22,23]. CAE and Maedi-visna are goat and sheep diseases that are internally notifiable, as defined in Appendix 1 of Romanian Order No. 79/2008 of the President of the National Veterinary Sanitary and Food Safety Authority (NSVFSA). However, there are no mandatory or voluntary SRLV control and eradication programs in Romania, which is a major obstacle in controlling the spread of the disease. Overall, the available information on the SRLV situation in Romania is very scarce. There are only a few studies about the prevalence of SRLVs in goats, in which a small number of herds and individual animals were investigated [24,25,26,27,28]. Therefore, the real epidemiological status of SRLVs in Romania is unknown. 

This study describes for the first time the *gag-pol* and *gag* sequences of SRLVs detected in Romanian sheep. Although cases of SRLV transmission from goats to sheep and vice versa have been noted [29,30,31,32,33,34,35] and many CAE outbreaks have been reported throughout Romania, and mixed flocks of sheep and goats are common in Romania [36], we did not find CAEV-like sequences. This may be due to the fact that the samples for testing were from the sheep showing chronic inflammatory changes in the lungs, which mainly occur with MVV infection [37]. Sequencing confirmed that the nested real-time PCR used in this study was able to correctly distinguish MVV from CAEV, as MVV-positive samples were only detected using primers and probes specific for MVV-like strains. All samples were negative using primers and probes to detect CAEV-like strains. Therefore, this test can be used instead of labor-intensive and costly SU5-ELISAs as an MVV/CAEV differentiation tool. More positive samples were detected using real-time than conventional PCR. Our study showed that 63 (51.6%) tested samples were positive via nested real-time PCR, while 31 (25.0%) and 24 (19.6%) samples were successfully amplified using *gag-pol* and *gag* primers, respectively. This may be due to the fact that nested real-time PCR is more sensitive than conventional PCR, and samples with very low SRLV proviral loads in genomic DNA could not be detected using conventional PCR. Another explanation could be the sequence variability of the Romanian strains. The primers used to amplify *gag* and *gag-pol* fragments may not work well on Romanian strains. The primers used in nested real-time PCR are new primers that are designed to anneal to highly conserved sequences located in the LTR and *gag*, which are currently selected after a thorough review of the 52 SRLV whole genome sequences available in the database. Therefore, these new designed primers may be more reliable. Moreover, studies on HIV have shown that the most conserved part of the HIV genome is not located in one of the open reading frames, but in the 5’ untranslated leader region [38]. Therefore, the LTR-*gag* fragment may be more suitable for the diagnosis of SRLVs than *gag* or *pol* fragments. However, this requires confirmation. On the other hand, our results revealed that only 52% of the samples tested in this study were positive for SRLVs using nested real-time PCR. Schaer et al. estimated that sensitivity of this nested real-time PCR was 75.5% [13]. Therefore, obtained results may suggest that the chronic inflammatory lesions in the lungs observed in some of tested sheep may be caused by other pathogens or may be related to SRLV compartmentalization. Undoubtedly, further knowledge on nucleotide sequences of SRLVs from different geographic regions may improve the sensitivity and specificity of PCRs. 

Molecular studies are based on different genomic regions of the virus, including *gag*, *gag-pol*, *pol*, *env* and LTR [4,5,6,7,8,9]. The *gag-pol* fragment is often used for phylogenetic analysis. Based on this fragment, SRLVs were classified into the following subtypes: A1–A5, A8–A9, A11, A19–A24, C, B1–B3 and E1–E2 [4]. The *gag-pol* phylogenetic tree and the pairwise genetic distances comparison revealed that Romanian sequences were closely related to A2 and A3 strains with mean genetic values that did not exceed 15% (12.0% and 11.5%). According to the criteria described by Shah et al. [39], sequences that differ by 15–27% represent distinct subtypes. Therefore, Romanian samples should be classified as A2/A3 strains. Our studies also revealed that A2 and A3 subtypes formed clusters without significant statistical support. The mean genetic distance between A2 and A3 subtypes was 12.6%, indicating that A2 and A3 strains belong to the same subtype. Therefore, it can be assumed that A2, A3 and Romanian sequences belong to one subtype. The mean genetic distance of all these strains was 10.2%, which supports this assumption. As described by Shah et al. [39], differences between A2 and A3 are often not large enough to separate these two subtypes. Our results support this finding. The results of this study also revealed that Romanian sequences differed from strains previously classified as A2/A3, which were detected in Spanish sheep [9,39,40]. In our phylogenetic tree, the Spanish A2/A3 sequences formed a separate cluster, and the mean genetic distances between these sequences and the A2 and A3 strains were 16.2% and 16.1%, respectively. In addition, the mean genetic distances between Spanish A2/A3 sequences and other subtypes representative for genotype A varied from 16.1% to 21.2%. Therefore, the Spanish A2/A3 strains may represent a new subtype based on *gag-pol* sequences.

To confirm the genetic assessment of the Romanian sequences, phylogenetic analysis was also performed using a shorter overlapping *gag* fragment (420 bp). As a result, it was possible to include subtypes A12, A13, A16, A17, A18, A25, A26 and A27 in the analysis. Consequently, sequences representing almost all subtypes detected so far in group A were used. Based on the *gag* fragment, the Romanian sequences belonged to genotype A, but could not be unambiguously assigned to the existing A subtype, as they were found to be equally related to the A3 and A17 subtypes. Moreover, the mean genetic distance between A3 and A17 subtypes was 10.5%. This may suggest that the differences between subtypes A3 and A17 may not be sufficient (especially when a highly conservative fragment is analyzed) to separate them as two different subtypes. Furthermore, obtained results may indicate that subtypes A3 and A17 and ovine Romanian SRLVs are phylogenetically linked. The similarity between the *gag* sequences of strains A17 and A3 was observed earlier by Olech et al. [41], although these subtypes formed separate clusters based on Bayesian model-based clustering. The inconclusive classification of Romanian strains indicates that future research should focus on more variable regions like *env*, since the *gag* region is highly conserved and retains less phylogenetic signals.

Our results revealed that classification of SRLVs into specific groups/subtypes can vary depending on the fragment analyzed. Many authors have also noted this phenomenon. For example, subtype A19 was defined based on partial *gag* sequence, but belonged to subtype B2 on the basis of *env* sequence [5]. Strain It009.2017 was defined as subtype A20 on the basis of the *gag* sequence, while on the basis of the *pol* sequence, this strain belonged to subtype A1 [19,41]. In addition, the LTR sequence of this strain showed the greatest similarity to strains belonging to subtype B1 [42]. Strains of subtype B5 were classified based on *pol* sequences, but were classified as B1 based on the *gag* region [33]. Furthermore, genotype D established on the basis of the *pol* sequence turned out to be genotype A on the basis of the *gag* sequence [11,43]. Olech et al., comparing *gag*, *env* and LTR fragments of the same Polish strains, also noted some discrepancies [42]. Therefore, more rigorous standards should be applied to provide correct classification of current and emerging strains. We suggest that phylogenetic analysis should be carried out on the basis of one specific fragment.

Analysis of genetic sequences of SRLVs is important not only for evaluating the spread of SRLV types and subtypes, but also for monitoring antigenic variation. It is known that antigenic variation can be responsible for the misdiagnosis of highly divergent subtypes, since no test is capable of detecting all circulating strains of SRLVs [11,44,45,46,47,48,49]. ELISA tests usually use the capsid protein as the antigen, so analysis of the epitope sequence of the *gag*-encoded protein is crucial. Our results revealed that Romanian sequences have similar epitope patterns to other existing subtypes. However, several observed mutations, especially E/K and R/K mutations in epitope 3 that were found only in Romanian strains, may have potential effect on antibody detection using standard serological techniques. This is especially relevant when competitive or blocking ELISAs, using monoclonal antibodies targeting specific epitopes, are commonly used.

## 5. Conclusions

In summary, this study describes, for the first time, the SRLV sequences detected in Romanian sheep and their relationship to known SRLV strains. The Romanian strains were closely related to the A2 and A3 strains on the basis of *gag-pol* fragment and to A3 and A17 subtypes based on *gag* fragment. The Romanian sequences showed some specific mutations in epitope 3, which may reflect their evolution. Further characterization of long, more variable sequences or full genome sequences and studies of recombination events would be useful to better understand the divergence of the Romanian SRLV strains. It will also be valuable to obtain SRLV sequences from other regions of Romania, from both sheep and goats. Knowledge of the genetic diversity of SRLVs and the genotypes/subtypes circulating in each country is important for epidemiological studies to better understand the evolution of these viruses and provide new information on the heterogeneity of SRLVs. Therefore, these studies filled a gap in SRLV research. The study points to the need for a new classification that addresses all cases of questionable clustering based on the current classification.

## Figures and Tables

**Table 1:** Information on *gag-pol* and *gag* SRLV sequences obtained from Romanian sheep.

Sample No.	Name	GenBank Accession Number	
		Gag-Pol	Gag
1.	RO20_Lung	OR671960	OR666886
2.	RO23_Lung	N/A	OR666885
3.	RO26_Lung	OR671963	OR666883
4.	RO27_Lung	OR671964	N/A
5.	RO29_Lung	OR671965	OR666882
6.	RO36_Lung	OR671959	OR666880
7.	RO45_Lung	OR671972	OR666876
8.	RO66_Lung	OR671975	OR666870
9.	RO70_Lung	N/A	OR666869
10.	RO75_Lung	OR671976	N/A
11.	RO82_Lung	OR671977	OR666868
12.	RO85_Lung	OR671978	OR666867
13.	RO23_Lymph node	OR671961	OR666884
14.	RO24_Lymph node	OR671962	OR666866
15.	RO34_Lymph node	OR671966	N/A
16.	RO35_Lymph node	OR671967	OR666881
17.	RO39_Lymph node	OR671968	N/A
18.	RO41_Lymph node	OR671969	OR666879
19.	RO42_Lymph node	OR671970	OR666878
20.	RO43_Lymph node	OR671971	OR666877
21.	RO45_Lymph node	N/A	OR666876
22.	RO46_Lymph node	OR671958	OR666874
23.	RO47_Lymph node	OR671973	OR666873
24.	RO48_Lymph node	N/A	OR666872
25.	RO52_Lymph node	OR671974	OR666871

N/A—not available.

**Table 2:** The mean genetic nucleotide distances (model p-distance) of the partial *gag* region of SRLV reference strains and Romanian SRLV strains obtained in this study. More than three strains were selected for each reference subtype, and evolutionary divergence was calculated based on the mean divergence of each set of subtypes and sequences obtained in this study.

	A1	A2	A3	A4	A5	A8	A9	A11	A12	A13	A16	A17	A18	A19	A20	A21	A22	A23	A24	A25	A26	A27
23L	13.1	11.8	8.4	14.2	10.8	16.1	12.2	15.2	10.5	11.3	13.9	9.0	11.9	16.1	14.5	13.3	18.7	15.7	12.3	11.9	11.8	11.8
20L	15.5	14.0	10.9	14.2	12.6	16.4	14.6	16.7	13.6	12.9	15.4	10.1	12.9	16.1	16.8	14.6	18.6	18.6	14.2	15.6	14.0	12.6
23LN	13.3	11.5	8.3	14.4	10.9	16.0	12.1	15.2	10.4	11.6	14.1	9.0	12.5	15.9	14.7	13.3	18.8	16.0	12.1	12.0	11.9	12.0
24LN	14.9	12.2	9.6	14.2	11.9	15.8	14.0	15.6	12.6	11.7	15.4	9.4	12.1	15.1	15.4	14.3	18.9	17.0	11.7	13.1	11.7	12.1
26L	15.7	12.2	10.6	14.7	12.4	16.4	14.1	15.7	12.5	13.0	16.5	8.3	13.3	18.7	15.9	14.3	19.6	18.5	12.8	14.5	11.1	11.8
27L	14.6	12.7	10.4	13.5	11.9	16.1	13.3	15.7	12.5	12.4	16.1	8.1	13.7	17.9	15.8	13.8	19.1	18.2	12.8	14.1	11.2	11.3
34LN	14.0	11.5	10.0	12.9	10.5	15.0	11.5	14.6	12.0	11.9	15.6	9.5	13.0	16.5	13.8	12.4	18.3	17.3	12.3	13.1	11.7	11.2
35LN	14.6	12.7	10.4	13.5	11.9	16.1	13.3	15.7	12.5	12.4	16.1	8.1	13.7	17.9	15.8	13.8	19.1	18.2	12.8	14.1	11.2	11.3
36L	15.7	12.2	10.6	14.7	12.4	16.4	14.1	15.7	12.5	13.0	16.5	8.3	13.3	18.7	15.9	14.3	19.6	18.5	12.8	14.5	11.1	11.8
39LN	13.0	10.0	8.3	11.3	9.1	14.2	11.0	13.9	10.9	10.6	13.6	8.3	12.0	15.2	12.8	12.0	17.2	15.7	11.3	11.5	11.0	10.3
41LN	15.9	12.6	10.4	15.2	12.5	17.8	14.2	16.5	12.5	12.5	16.1	10.3	13.8	17.1	16.6	13.1	20.1	18.5	13.0	13.7	13.1	13.2
42LN	15.5	14.0	10.9	15.2	12.6	16.4	14.6	16.7	13.6	12.9	15.4	10.1	12.9	16.1	16.8	14.6	18.6	18.6	14.2	15.6	14.0	12.6
43LN	14.6	12.7	10.4	13.5	11.9	16.1	13.3	15.7	12.5	12.4	16.1	8.1	13.7	17.9	15.8	13.8	19.1	18.2	12.8	14.1	11.2	11.3
45L	15.1	12.7	9.1	14.6	12.0	16.1	13.3	15.0	11.6	11.5	15.7	8.1	11.9	17.6	15.9	13.0	18.9	17.5	12.3	13.9	11.2	10.7
45LN	15.7	12.2	10.6	14.7	12.4	16.4	14.1	15.7	12.5	13.0	16.5	8.3	13.3	18.7	15.9	14.3	19.6	18.5	12.8	14.5	11.1	11.8
46LN	15.7	12.2	10.6	14.7	12.4	16.4	14.1	15.7	12.5	13.0	16.5	8.3	13.3	18.7	15.9	14.3	19.6	18.5	12.8	14.5	11.1	11.8
47LN	14.6	12.8	9.7	14.0	11.5	14.9	13.9	16.0	12.2	12.4	15.8	9.4	14.5	16.6	14.9	13.5	18.6	18.6	13.3	13.5	12.2	10.8
66L	14.4	13.8	10.7	14.5	10.4	16.5	14.0	16.2	12.1	11.3	15.4	11.4	14.9	14.9	15.5	14.0	18.6	17.5	13.3	13.6	12.5	13.0
70L	16.5	13.1	11.4	14.9	12.6	16.3	14.7	15.5	12.3	13.2	15.6	11.0	14.2	16.4	14.6	14.7	20.0	16.9	13.9	12.7	13.1	13.2
82L	16.0	12.2	9.4	13.5	11.9	15.6	14.0	14.5	11.7	11.9	15.8	9.9	13.3	16.0	15.8	13.2	17.4	16.5	12.5	13.3	11.4	11.9
85L	16.3	10.4	9.1	15.1	13.8	16.2	13.3	13.7	12.3	12.1	16.2	9.8	13.0	16.3	16.5	15.1	20.7	16.0	12.8	13.1	11.9	12.4
75L	14.8	10.5	9.4	14.4	11.9	15.3	12.0	15.2	12.1	11.3	15.4	8.5	11.4	14.3	13.6	14.9	19.0	15.9	12.7	10.6	11.9	10.6
29L	14.3	12.6	10.9	14.6	10.8	15.6	12.7	16.0	12.3	11.5	16.2	8.3	15.2	16.0	15.9	13.9	19.3	18.3	12.8	14.8	12.0	12.4
48LN	16.9	13.6	9.9	16.4	13.2	18.4	15.8	16.3	14.4	14.9	15.7	12.7	14.7	17.7	16.5	14.2	19.1	17.0	15.7	14.0	16.0	14.6
52LN	15.6	10.6	9.5	14.9	11.8	16.6	13.1	15.8	12.4	11.8	15.8	9.4	13.3	16.4	15.8	14.0	17.7	17.9	14.9	12.8	13.3	12.5

L—lung, and LN—lymph node.

## Data Availability

All data generated and analyzed in this study are included in this article.

## Supplementary Materials

The following supporting information can be downloaded at https://www.mdpi.com/article/10.3390/ani13233718/s1, Table S1: Estimated mean genetic nucleotide distances (model p-distance) between subtypes of genotype A and Romanian strains based on the *gag-pol* fragment; Table S2: Estimated mean genetic nucleotide distances (model p-distance) between subtypes of genotype A and Romanian strains based on the CA fragment of *gag* gene.
