# Supplementary material for: First Molecular Characterization of Small Ruminant Lentiviruses Detected in Romania
Source: Animals (Basel). 2023 Nov 30;13(23):3718. doi: 10.3390/ani13233718 (PMC10705781; doi:10.3390/ani13233718)
Supplement: Supplementary file 1 [file animals-13-03718-s001.zip › animals-2731281-supplementary/Table S1.pdf]

Table S1. Estimated of mean genetic nucleotide distances (model p-distance) between subtypes of genotype A and Romanian strains based on the *gag-pol* fragment.

	A1	A2	A2/A3	A3	A4	A5	A8	A9	A11	A19	A20	A21	A22	A23	A24
A1	-	-	-	-	-	-	-	-	-	-	-	-	-	-	-
A2	17.0	-	-	-	-	-	-	-	-	-	-	-	-	-	-
A2/A3	17.5	16.2	-	-	-	-	-	-	-	-	-	-	-	-	-
A3	17.1	12.6	16.1	-	-	-	-	-	-	-	-	-	-	-	-
A4	18.0	17.8	17.3	17.3	-	-	-	-	-	-	-	-	-	-	-
A5	16.4	15.9	16.6	14.5	15.6	-	-	-	-	-	-	-	-	-	-
A8	18.8	18.0	17.9	17.3	18.8	18.9	-	-	-	-	-	-	-	-	-
A9	19.8	18.0	18.1	17.0	17.5	18.1	17.9	-	-	-	-	-	-	-	-
A11	19.2	17.2	18.0	16.7	20.0	17.3	17.5	16.9	-	-	-	-	-	-	-
A19	20.1	18.3	18.1	17.4	18.8	17.3	18.4	17.0	17.0	-	-	-	-	-	-
A20	18.7	18.5	18.8	17.4	19.6	17.3	19.7	19.6	18.9	21.8	-	-	-	-	-
A21	18.1	15.4	16.4	15.1	18.5	16.2	18.1	18.0	17.1	19.1	18.5	-	-	-	-
A22	22.3	20.2	21.2	20.0	21.1	19.1	21.7	21.1	21.0	21.4	23.0	20.5	-	-	-
A23	19.5	18.0	18.6	17.6	19.4	18.6	18.6	18.4	17.6	17.8	18.6	19.0	21.1	-	-
A24	17.7	15.7	17.1	15.4	18.0	15.9	16.1	16.1	16.0	16.5	19.4	16.7	20.8	17.5	-
Romanian strains	**16.8**	**12.0**	**15.6**	**11.5**	**16.7**	**14.2**	**17.1**	**17.5**	**17.0**	**17.2**	**18.5**	**15.2**	**19.5**	**17.7**	**15.0**
