# Supplementary material for: First Molecular Characterization of Small Ruminant Lentiviruses Detected in Romania
Source: Animals (Basel). 2023 Nov 30;13(23):3718. doi: 10.3390/ani13233718 (PMC10705781; doi:10.3390/ani13233718)
Supplement: Supplementary file 1 [file animals-13-03718-s001.zip › animals-2731281-supplementary/Table S2.pdf]

Table S2. Estimated of mean genetic nucleotide distances (model p-distance) between subtypes of genotype A and Romanian strains based on the CA fragment of *gag* gene.

	A1	A2	A2/A3	A3	A4	A5	A8	A9	A11	A12	A13	A16	A17	A18	A19	A20	A21	A22	A23	A24	A25	A26	A27	Romanian strains
A1		0,014	0,012	0,015	0,015	0,014	0,015	0,016	0,015	0,014	0,015	0,014	0,015	0,016	0,014	0,015	0,014	0,017	0,016	0,016	0,015	0,016	0,014	0,013
A2	0,165		0,011	0,014	0,015	0,013	0,014	0,015	0,014	0,012	0,012	0,013	0,013	0,013	0,014	0,014	0,012	0,015	0,014	0,013	0,012	0,013	0,013	0,011
A2/A3	0,164	0,146		0,012	0,012	0,011	0,012	0,012	0,012	0,010	0,011	0,012	0,012	0,013	0,011	0,013	0,011	0,014	0,013	0,012	0,011	0,012	0,012	0,010
A3	0,160	0,137	0,143		0,015	0,013	0,014	0,014	0,013	0,012	0,014	0,015	0,012	0,016	0,013	0,015	0,013	0,016	0,014	0,013	0,014	0,015	0,014	0,010
A4	0,165	0,161	0,156	0,161		0,014	0,014	0,016	0,015	0,014	0,015	0,013	0,014	0,017	0,014	0,015	0,015	0,016	0,015	0,014	0,014	0,015	0,015	0,013
A5	0,169	0,153	0,148	0,141	0,155		0,013	0,014	0,014	0,012	0,014	0,014	0,013	0,015	0,013	0,013	0,013	0,015	0,014	0,013	0,014	0,014	0,013	0,010
A8	0,172	0,174	0,168	0,164	0,171	0,172		0,013	0,013	0,013	0,014	0,014	0,014	0,015	0,012	0,014	0,014	0,016	0,013	0,011	0,013	0,014	0,014	0,012
A9	0,164	0,157	0,145	0,137	0,157	0,152	0,149		0,013	0,014	0,015	0,016	0,014	0,017	0,013	0,015	0,015	0,017	0,014	0,013	0,015	0,016	0,015	0,013
A11	0,174	0,158	0,158	0,152	0,178	0,171	0,166	0,148		0,012	0,013	0,014	0,014	0,015	0,011	0,013	0,013	0,016	0,012	0,013	0,014	0,014	0,013	0,013
A12	0,167	0,130	0,145	0,130	0,164	0,151	0,165	0,165	0,147		0,012	0,013	0,012	0,014	0,013	0,012	0,012	0,015	0,013	0,012	0,012	0,013	0,013	0,010
A13	0,163	0,113	0,145	0,132	0,151	0,147	0,164	0,160	0,143	0,133		0,014	0,015	0,014	0,014	0,014	0,012	0,017	0,015	0,013	0,013	0,014	0,012	0,011
A16	0,184	0,159	0,157	0,175	0,155	0,167	0,191	0,194	0,181	0,166	0,160		0,014	0,014	0,014	0,014	0,013	0,015	0,015	0,014	0,013	0,014	0,013	0,012
A17	0,155	0,128	0,147	0,105	0,142	0,133	0,162	0,126	0,160	0,123	0,137	0,157		0,015	0,013	0,014	0,014	0,015	0,015	0,013	0,014	0,014	0,013	0,009
A18	0,188	0,128	0,147	0,154	0,171	0,170	0,187	0,173	0,175	0,154	0,128	0,166	0,141		0,015	0,015	0,014	0,015	0,016	0,015	0,014	0,014	0,014	0,013
A19	0,182	0,173	0,157	0,147	0,165	0,165	0,162	0,133	0,148	0,168	0,172	0,191	0,157	0,183		0,013	0,014	0,015	0,012	0,012	0,013	0,014	0,014	0,012
A20	0,181	0,157	0,174	0,170	0,181	0,159	0,177	0,179	0,158	0,155	0,158	0,170	0,169	0,175	0,196		0,013	0,015	0,014	0,014	0,013	0,013	0,013	0,012
A21	0,159	0,153	0,144	0,144	0,188	0,157	0,182	0,166	0,155	0,143	0,139	0,169	0,160	0,155	0,185	0,159		0,015	0,015	0,013	0,013	0,013	0,012	0,011
A22	0,233	0,206	0,205	0,193	0,214	0,191	0,219	0,209	0,217	0,205	0,210	0,226	0,194	0,196	0,205	0,216	0,203		0,015	0,015	0,016	0,016	0,015	0,014
A23	0,203	0,177	0,186	0,169	0,186	0,181	0,176	0,158	0,151	0,169	0,185	0,205	0,175	0,201	0,156	0,168	0,198	0,209		0,013	0,015	0,015	0,015	0,013
A24	0,163	0,138	0,148	0,136	0,159	0,150	0,138	0,133	0,148	0,141	0,140	0,177	0,137	0,166	0,140	0,174	0,148	0,202	0,154		0,013	0,013	0,013	0,011
A25	0,162	0,122	0,148	0,142	0,153	0,151	0,162	0,142	0,164	0,140	0,122	0,157	0,138	0,125	0,163	0,148	0,158	0,205	0,180	0,151		0,013	0,013	0,012
A26	0,164	0,117	0,141	0,142	0,161	0,137	0,153	0,157	0,142	0,134	0,113	0,162	0,137	0,111	0,166	0,142	0,136	0,179	0,180	0,126	0,111		0,013	0,011
A27	0,150	0,129	0,145	0,136	0,159	0,141	0,169	0,156	0,149	0,139	0,114	0,151	0,142	0,132	0,173	0,153	0,135	0,200	0,183	0,149	0,129	0,114		0,011
Romanian strains	0,156	0,126	0,137	0,103	0,144	0,125	0,166	0,138	0,157	0,126	0,124	0,160	0,100	0,136	0,160	0,162	0,142	0,196	0,178	0,131	0,138	0,121	0,123	
